# Genome-wide association study to identify genomic regions influencing spontaneous fertility in maize haploids

**DOI:** 10.1007/s10681-019-2459-5

**Published:** 2019-07-08

**Authors:** Vijay Chaikam, Manje Gowda, Sudha K. Nair, Albrecht E. Melchinger, Prasanna M. Boddupalli

**Affiliations:** 1 International Maize and Wheat Improvement Center (CIMMYT), ICRAF Campus, UN Avenue, Gigiri, P.O. Box 1041–00621, Nairobi, Kenya; 2 International Maize and Wheat Improvement Center (CIMMYT), ICRISAT Campus, Patancheru, Greater Hyderabad, 502324 India; 3 Institute of Plant Breeding, Seed Science and Population Genetics, University of Hohenheim, 70593 Stuttgart, Germany

**Keywords:** Doubled haploid (DH), Haploid male fertility (HMF), Haploid female fertility (HFF), Haploid fertility (HF), Genome-wide association study (GWAS), Genomic prediction (GP)

## Abstract

Efficient production and use of doubled haploid lines can greatly accelerate genetic gains in maize breeding programs. One of the critical steps in standard doubled haploid line production is doubling the haploid genome using toxic and costly mitosis-inhibiting chemicals to achieve fertility in haploids. Alternatively, fertility may be spontaneously restored by natural chromosomal doubling, although generally at a rate too low for practical applications in most germplasm. This is the first large-scale genome-wise association study to analyze spontaneous chromosome doubling in haploids derived from tropical maize inbred lines. Induction crosses between tropicalized haploid inducers and 400 inbred lines were made, and the resulting haploid plants were assessed for haploid male fertility which refers to pollen production and haploid fertility which refers to seed production upon self-fertilization. A small number of genotypes were highly fertile and these fertility traits were highly heritable. Agronomic traits like plant height, ear height and tassel branch number were positively correlated with fertility traits. In contrast, haploid induction rate of the source germplasm and plant aspect were not correlated to fertility traits. Several genomic regions and candidate genes were identified that may control spontaneous fertility restoration. Overall, the study revealed the presence of large variation for both haploid male fertility and haploid fertility which can be potentially exploited for improving the efficiency of doubled haploid derivation in tropical maize germplasm.

**Electronic supplementary material:**

The online version of this article (10.1007/s10681-019-2459-5) contains supplementary material, which is available to authorized users.

## Introduction

Haploids have become very important in maize breeding as a source of completely homozygous inbred lines, referred to as doubled haploid (DH) lines. Use of DH lines in maize breeding increases the genetic gain by shortening the breeding cycle time, enables cost savings and increases the efficiency and precision of selection (Liu et al. 2016; Prasanna 2012; Xu et al. 2017). Haploids are generated in vivo in maize by pollinating the source germplasm with pollen from maternal haploid inducers (Chaikam 2012). The resulting seeds/seedlings are sorted based on various markers (Chaikam et al. 2016; Chase and Nanda 1965; Melchinger et al. 2014), and the selected haploid seed (D_0_ seed) is generally used in DH production. Due to misclassification or inhibition of markers used for classification of induction cross seed, the resulting D_0_ seed usually includes false positive (hybrid) seed, which are later removed in the DH nursery based on their phenotype (Mahuku 2012; Prigge and Melchinger 2012). The true haploids in the DH nursery may be chimeras containing homozygous diploid cells but are still referred to as haploids because of predominance of haploid cells and tissues. DH lines are produced by self-pollinating the fertile haploid plants (Chaikam and Mahuku 2012; Prigge and Melchinger 2012).

In general, haploid plants are expected to be completely sterile as meiotic cell divisions cannot proceed normally in the haploid sporocytes, resulting in non-formation of the male and female gametophytes and gametic cells (Chaikam and Mahuku 2012). Maize breeding programs generally rely on artificial chromosome doubling protocols involving mitotic inhibitor chemicals for achieving fertility in haploid plants (Chaikam and Mahuku 2012; Prigge and Melchinger 2012). In the literature, haploid fertility (HF) generally refers to production of at least one seed from a haploid plant upon self-fertilization (Kleiber et al. 2012). HF comprises haploid male fertility (HMF) and haploid female fertility (HFF) (Ren et al. 2017). The objective of artificial chromosomal doubling protocols is to achieve genome duplication in the shoot apical meristem that gives rise to male and female inflorescences, thereby restoring the fertility in haploid plants. Most of the current genome doubling protocols are based on the antimitotic chemical colchicine (Chaikam and Mahuku 2012; Chalyk 2000; Gayen et al. 1994; Liu et al. 2016; Prigge and Melchinger 2012) which disrupts normal function of the mitotic spindle by binding to the microtubule subunit protein tubulin and preventing microtubule polymerization (Sackett and Varma 1993; Taylor 1965). This temporarily arrests the cells in metaphase, delays the centromere division and migration of sister chromatids to opposite poles, which brings all chromosomes into the same nuclei after centromere division, and ultimately leads to a cell with duplicated chromosomes (Levan 1938). The effect of colchicine on the spindle is reversible; therefore, whenever the colchicine effect is reduced or nil, normal mitosis will continue in the cells (Levan 1938).

Colchicine, even though a very effective chromosomal doubling agent, is toxic to humans (Finkelstein et al. 2010). In addition, colchicine is also hazardous to the environment, and hence needs to be disposed properly after use (Melchinger et al. 2016). Colchicine is an expensive chemical, and the establishment of facilities for large-scale germination of haploid seeds, treatment of haploid seedlings with colchicine, recovery of D_0_ seedlings, colchicine waste storage and disposal, further increase the expenses. In addition, artificial chromosome doubling involves several labor-intensive steps such as germination, chromosomal doubling treatment and transplanting of haploids. Moreover, there is a risk of losing considerable proportion of haploids after colchicine treatment. Recently, research has been undertaken for developing chromosome doubling protocols based on less toxic mitotic herbicides (Melchinger et al. 2016) and nitrous oxide gas (Kato and Geiger 2002; Molenaar et al. 2018). However, these less toxic alternatives still need suitable facilities and labor requirements, similar to colchicine treatment.

A possible alternative for artificial chromosomal doubling in haploid plants is to rely on spontaneous chromosome doubling, where both the male and female reproductive organs produce fertile gametes without application of any artificial chromosomal doubling agents. Studies have indicated that some haploids naturally exhibit a certain degree of HMF and HFF and this fertility was ascribed to sectors of cells in reproductive organs with spontaneously doubled chromosomes (Chase 1949; Ma et al. 2018). Since a high proportion of untreated haploid plants (~ 97–100%) showed seed set on ears that were pollinated with pollen from normal diploid plants (Chalyk 1994; Geiger et al. 2006), it is believed that HFF is not a limitation for production of DH lines. However, these studies also indicated that most haploid plants are male sterile (Chalyk 1994; Geiger et al. 2006). Hence, HMF is generally considered as a limiting factor in production of DH lines (Chalyk 1994; Kleiber et al. 2012; Ren et al. 2017; Wu et al. 2017). Recent studies based on broader germplasm and large number of haploid plants indicated that HMF is genotype dependent (Geiger and Schönleben 2011; Kleiber et al. 2012; Ma et al. 2018; Wu et al. 2017). HMF is also higher under favorable conditions like greenhouses (Kleiber et al. 2012) and can vary greatly from few anthers producing pollen to complete tassel fertility. However, on average, the rate of spontaneous HF is about 20-fold less than HF due to artificial doubling. Furthermore, spontaneously doubled haploid plants produce less seed than artificially doubled haploid plants (Kleiber et al. 2012).

Considering the challenges in artificial chromosomal doubling, improved natural HF can enhance the efficiency of DH lines production with significant reduction in costs involved in germination, seedling care, chromosomal doubling and transplanting. Hence, identifying genotypes with high spontaneous genome duplication rates in elite germplasm, and identifying the genomic regions contributing favorably to HMF and HF is important in maize breeding programs.

A recent study on HMF indicated that it is controlled by two or more major genes with additive effects (Wu et al. 2017). Using bi-parental mapping populations constituting parents with low and high HMF, four quantitative trait loci (QTL) affecting the HMF were identified and a major QTL was fine-mapped (Ren et al. 2017). However, there were no publications so far on the genetic architecture of HF. The objectives of this study are to (1) investigate the available genetic variation for HMF and HF in a large set of elite tropical maize breeding lines, (2) identify the elite tropical inbred lines with high rates of natural HMF and HF, and (3) to identify the genomic regions influencing HMF and HF using GWAS.

## Materials and methods

### Genetic materials and haploid induction

A panel of 400 inbred lines were used in this study to generate haploid seeds and to study spontaneous haploid fertility. This panel included 188 CIMMYT inbred lines that were part of the Drought Tolerant Maize for Africa (DTMA) association mapping panel, and the rest were CIMMYT Maize Lines (CMLs) adapted to tropics/subtropics across Latin America, sub-Saharan Africa and Asia. Haploid induction and evaluation of haploid fertility traits were carried out in 2 years. Haploid induction crosses were produced at Agua Fria experimental station in Mexico (20.26°N, 97.38°W; ~ 110 m elevation). Inbred lines were grown in two replications with each plot consisting of two rows of 4.5 m with 19 plants/row. Two tropicalized haploid inducers, TAIL8 and TAIL9 were used as a pollen parents for haploid induction crosses (Chaikam et al. 2016). Haploid inducers were stagger-planted four times at a five-day interval to make enough pollen available for the inbreds from different maturity groups. All plants from the source germplasm (inbred lines) were pollinated with bulked pollen from the inducers. Ears from each of the induced inbred lines were harvested at physiological maturity.

### Experimental design and trait assessment

Seeds from induction crosses were planted in two years at CIMMYT’s Agua Fria experimental station to identify haploids and to assess the fertility traits. Even though the haploid inducers used in this study are equipped with *R1*-*nj* marker for haploid seed identification, it was not used for separation of haploid and diploid seeds, as the expression of the *Navajo* phenotype conditioned by *R1*-*nj* could be potentially inhibited in a significant proportion of tropical inbreds (Chaikam et al. 2015). All the seeds lines resulting from the induction crosses were evaluated in replicated trials in an alpha-lattice design and for both the inducers 15% of the entries were common. Field was prepared into beds spaced at 75 cm and separated by furrows. Unsorted seeds from induction crosses were planted in two rows at both sides of the bed with 10 cm of inter-row space. For each entry, a minimum of 1000 seeds resulting from the induction cross were used for planting. Three to four weeks after planting, each survived plant was assessed for plant vigor, leaf erectness, and paleness of leaves to differentiate haploids from the diploids. Haploid plants typically show poor vigor, erect leaves and pale leaves compared to the diploids (Chaikam et al. 2016; Melchinger et al. 2013) (Supplementary Fig S1). From the total number of survived plants, the number of haploids and number of diploids were recorded for each entry. All the diploid plants were removed from the field and the surviving haploid plants were grown under good agronomic management. Any plant, whose ploidy could not be definitively established at this stage was left in the field till anthesis, by which stage the plant characteristics becomes more obvious to differentiate haploids from diploids. We have also recorded other agronomic traits like plant height, ear height, and number of tassel branches as an average of 10 haploid plants for each entry in each replication, as described earlier by Chaikam et al. (2016). Plant aspect was visually scored on a scale of 1–5 where score 1 indicates uniform plants with agronomically desirable traits, while score 5 indicates non-uniform plants with agronomically non-desirable traits.

The ear of each haploid plant was covered with a shoot bag before the emergence of silks. HMF was assessed based on anther emergence and pollen shedding. At anthesis stage, each haploid plant was visually assessed for anther emergence. Tassels with emerged anthers were bagged with white wax coated glassine bags early in the morning. Two to three hours later, the tassels were shaken, and pollen was collected into glassine bags. Presence of pollen in the glassine bags was visually assessed. Plants with extruded anthers and visible pollen grains were self-pollinated thrice on three consecutive days. All the pollinated ears were harvested manually and assessed visually for seed set. None of the ears harvested showed *R1*-*nj* marker expression indicating that the ears were derived from true haploids. The number of haploid plants with seeds was counted for each entry in each replication.

### Phenotypic data analysis

The inbreds with more than 25 haploids per replication was included for further data analysis. This reduced the final number of inbreds used in the analysis to 315. Haploid induction rate (HIR) was calculated as the proportion of total number of haploids in the total number of survived plants and expressed in percentage. Non-germinated seed and seedlings/plants that died before evaluation of ploidy status were not considered in HIR determination. HMF was calculated as a proportion of the total number of pollen-producing haploid plants from the total number of haploid plants per plot and expressed in percentage. HF was calculated by dividing the total number of seed-producing haploids from the total number of haploid plants per plot and expressed in percentage. Analyses of phenotypic data revealed normality in the distribution of residuals for all traits except for HMF and HF, where data was skewed more towards zero. Therefore, HMF and HF data were transformed by applying a logit-transformation.

Analysis of variance for each trait was carried out using the PROC MIXED procedure with restricted maximum likelihood (REML) option in SAS 9.2 (SAS Institute 2010). Variance components across environments were determined by following linear mixed model:

$$ {\text{Y}}_{\text{ijko}} =\upmu + {\text{G}}_{\text{i}} + {\text{E}}_{\text{j}} + {\text{GE}}_{\text{ij}} + {\text{I}}_{\text{k}} + {\text{r}}_{\text{l}} + {\text{b}}_{\text{ol}} + {\text{e}}_{\text{ijklo}} , $$

where Y_ijklo_ is the observed phenotype in the *j*th environment at *l*th replication of the *o*th incomplete block for the haploid from the cross of the *i*th genotype with the *k*th inducer*. μ* is an intercept term, r_*l*_ is the effect of the *l*th replication, b_*ol*_ is the effect of the *o*th incomplete block in the *l*th replication, G_*i*_ is the genetic effect of the *i*th genotype, E*j* is the effect of the *jth* environment, and e_*ijklo*_ is the experimental error. Firstly, the replication, inducer and genotype effects were taken as fixed and genotype x environment interactions (GxE) and incomplete block effect as random to obtain Best linear unbiased estimates (BLUEs) for each line. Secondly, the effects of genotype, GxE and incomplete blocks were treated as random to estimate their variances and the residual error variance (σ_G_^2^, σ_GxE_^2^, σ_b_^2^, and σ_e_^2^, respectively). Heritability (*h*^*2*^) for each trait was calculated as *h*^*2*^ = σ_G_^2^/(σ_G_^2^ + (σ_GxE/E_^2^) + (σ_e_^2^/Exr)) where E and r refers to the number of environments and replications, respectively. Phenotypic correlations among traits were calculated using R (R Core Team 2018).

### Genotyping and quality control

DNA of all inbred lines planted in the haploid induction nurseries was extracted from the leaf samples of 3–4 week-old seedlings using the standard CIMMYT laboratory protocol (CIMMYT 2001). Genotyping was carried out using the Genotyping-by-Sequencing (GBS) platform (Elshire et al. 2011) at the Institute for Genomic Diversity, Cornell University, Ithaca, USA following the procedure described by Elshire et al. (2011). Briefly, genomic DNA was digested with the restriction enzyme *Ape*KI. GBS libraries were constructed in 96-plex and sequenced on Illumina HiSeq 2000 (Elshire et al. 2011). Single nucleotide polymorphism (SNP) calling was performed using the TASSEL (Trait Analysis by aSSociation Evolution and Linkage) GBS Pipeline, and a GBS 2.7 TOPM (tags on physical map) file was used to anchor reads to the Maize B73 RefGen_v2 reference genome (Glaubitz et al. 2014). For quality screening, SNPs which were either monomorphic, had a call rate < 0.09, heterozygosity of > 0.05, or had a minor allele frequency (MAF) of < 0.05 were discarded from the analysis. After these quality checks, 214,520 high quality SNPs were retained for GWAS. For principal component analysis (PCA) and kinship matrix, high quality SNPs MAF ≥ 0.05 were used, whereas for analysis of linkage disequilibrium (LD) between adjacent markers, SNPs were filtered for MAF ≥ 0.30.

### PCA, Kinship and LD analysis

Two-dimensional plot of the first two principal components (PC) was drawn to visualize the possible population stratification among the inbred lines. A kinship matrix was also computed from identity-by-state (IBS) distances matrix (as executed in TASSEL). The extent of genome-wide LD was based on pairwise r^2^ values between adjacent SNPs among the high-quality SNPs and physical distances between these SNPs (Remington et al. 2001). Genome-wide LD across 36,158 SNPs was investigated. Nonlinear models with r^2^ as dependent variable and physical distances as independent variable were fitted into the genome-wide and chromosome-wise LD data using the ‘nlin’ function in R (R core team 2014). Average pairwise distances in which LD decayed at r^2^ = 0.2; r^2^ = 0.1 values were then calculated based on the model given by Remington et al. (2001).

### GWAS

GWAS was conducted by regressing BLUEs of inbred lines on marker genotypes by using mixed linear model (MLM) (Yu et al. 2006) which accounts for population structure and kinship, in TASSEL (Bradbury et al. 2007), version 5.2.24. As population structure can result in spurious associations, it was considered by using the first five PCs which together contributed > 14% of the total variation. A vector of random effects with covariance structure given by the kinship matrix was used to account for the degree of relatedness. The MLM was run with the optimum level of compression. Genome-wide scans for marker–trait associations were conducted to detect main-effect QTL. The genome-wide threshold for marker-trait associations was set at *P* < 0.05 using the Bonferroni-Holm procedure (Zhang et al. 2010). The 50 bp source sequences of the significantly associated SNPs were used to perform BLAST searches against the ‘B73’ RefGen_v2 (http://blast.maizegdb.org/home.php?a=BLAST_UI). Within the local LD block including associated SNPs, the filtered genes in MaizeGDB (http://www.maizegdb.org) containing directly or adjacent to each associated SNP were considered as possible candidate genes for HMF and/or HF.

Genomic prediction (GP) was performed by Ridge Regression-Best Linear Unbiased Prediction (rrBLUP) employing the R package ‘rrBLUP’ with five-fold cross-validation (Endelman 2011). From the GBS data, a sub-set of 4580 SNPs distributed uniformly across genome, with no missing values, and minor allele frequency > 0.05 were used for GP in the present panel. The prediction accuracy was estimated as the correlation coefficient between genomic estimated breeding values (GEBVs) and the observed phenotypes divided by the square root of the heritability (Dekkers 2007). The sampling of training and validation sets was repeated 100 times.

## Results

Analyses of phenotypic data revealed normality in the distribution of residuals for all agronomic traits except for HMF and HF, where data was skewed more towards zero. Therefore, HMF and HF data were transformed by applying a logit-transformation. After the estimation of variance components and BLUEs, the BLUEs data were back transformed and used for further analyses. HMF and HF were assessed in a final set of 315 inbred lines which revealed large genetic variation for both traits (Table 1 and Supplementary Fig S2). Across all the lines, HMF was averaged at 15.65% and ranged from 0.61 to 77.6%, whereas HF ranged from 0.40 to 70.01% with a mean of 5.55%. ANOVA revealed significant genotypic and GxE variances for HMF, whereas only genotypic variances were significant for HF. Heritabilities were high for both HMF and HF. In addition, all the agronomic traits measured on haploids also showed significant genotypic variances with high heritability. Among the 315 lines evaluated for HMF and HF, 11 lines with > 50% HMF and > 10% HF were identified as promising sources for spontaneous haploid fertility (Table 2). The line DTMA-159 showed the highest HMF (77%) and HF (70%), followed by DTMA-59 which showed 63.9% HMF and 53.6% HF (Table 2).

**Table 1:** Mean, range, and components of variance for haploid male fertility and related traits for maize inbred line association mapping panel

	HMF	HF	HIR	Pasp	Pht	Eht	Tbr
Mean	15.65	5.55	7.68	2.53	52.57	15.85	5.21
Min	0.61	0.43	6.53	1.15	25.17	7.99	1.08
Max	77.60	70.01	12.58	4.10	81.44	26.85	17.75
σ_G_^2^	0.22**	0.15**	1.05**	0.35**	84.69**	16.76**	8.52**
σ_GxE_^2^	0.03*	0.00	0.12**	0.00	6.89**	0.00	0.00
σ_e_^2^	0.11	0.09	1.36	0.16	20.87	11.91	1.25
*h* ^*2*^	0.84	0.87	0.72	0.90	0.91	0.85	0.96
LSD	0.21	0.14	1.08	0.25	18.11	7.48	1.62
CV	39.13	77.05	15.21	15.80	8.68	21.73	21.77

*HMF* haploid male fertility (in %), *HF* haploid fertility (in %), *HIR* haploid induction rate (in %), *Pasp* plant aspect (on a 1–5 scale), *Tbr* number of tassel branches, *Pht* plant height, *Eht* ear height

*, **Significant at *P *< 0.01 and *P *< 0.05 level

**Table 2:** List of maize inbred lines with high levels of spontaneous haploid male fertility (HMF) and haploid fertility (HF)

Genotype	HMF (%)	HF (%)
DTMA-159	77.60	70.01
DTMA-99	74.37	49.16
DTMA-261	72.52	15.50
CML364	71.30	50.24
DTMA-64	65.21	29.04
DTMA-59	63.98	53.66
DTMA-128	61.02	26.46
DTMA-20	59.25	21.53
CML435	55.69	12.89
DTMA-122	53.48	26.60
DTMA-197	52.64	24.39

HMF was significantly correlated with plant height, ear height and number of tassel branches (Table 3). However, plant aspect showed no significant correlation with HMF. As expected, HMF was significantly correlated with HF. Like HMF, HF was significantly correlated with plant height, ear height and number of tassel branches, but no significant correlation was observed with plant aspect. Both HMF and HF were not significantly correlated with HIR of the inbred lines. Plant aspect is significantly and negatively correlated with tassel branches, plant height, and ear height. Number of tassel branches is significantly correlated with both plant height and ear height.

**Table 3:** Genetic correlations among different traits evaluated in the panel

Traits	HMF	HF	HIR	Pasp	Tbr	Pht
HF	0.77**					
HIR	0.02	0.05				
Pasp	0.02	− 0.06	− 0.22**			
Tbr	0.16*	0.17*	0.05	− 0.41**		
Pht	0.17*	0.17*	0.03	− 0.67**	0.36**	
Eht	0.20**	0.25**	− 0.16*	− 0.58**	0.22**	0.69**

For trait abbreviations, see Table 1

*, **Significant at *P *< 0.05 and 0.01 level, respectively

A total of 955,690 SNP markers were obtained from the GBS platform. SNPs were initially filtered to remove SNPs with missing rate > 10%, SNPs with minor allele frequency (MAF) < 5% and heterogeneity > 5%, resulting in 214,520 markers used for GWAS analyses, these genotypic data are available in CIMMYT’s data repository: http://hdl.handle.net/11529/10431. Per chromosome, the average marker heterogeneity was approximately 0.037, the proportion of missing values was close to 0.05, and the minor allele frequency (MAF) was around 0.21, respectively (Supplementary Fig S3).

**Fig. 1:**
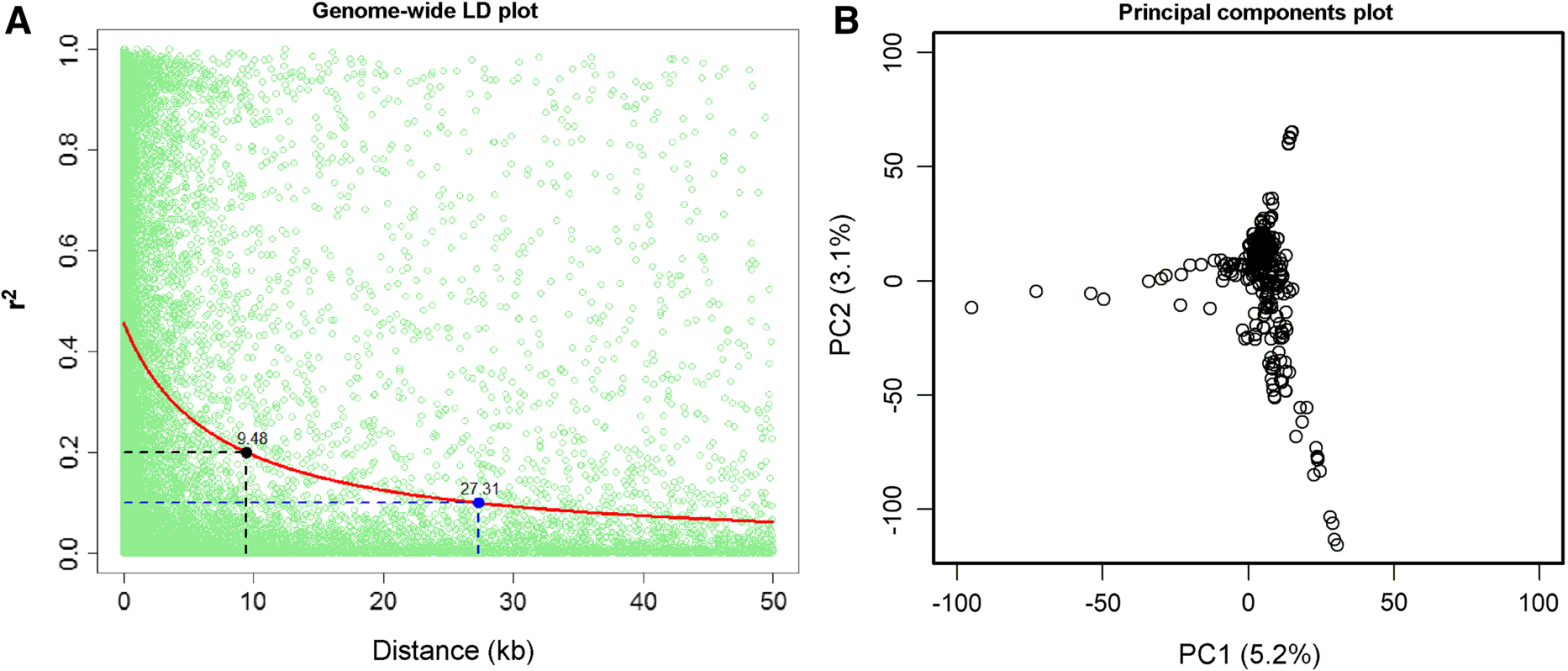
**A** Linkage disequilibrium (LD) plot representing the average genome-wide LD decay in the panel with genome-wide markers. The values on the Y-axis represents the squared correlation coefficient r^2^ and the X-axis represents the physical distance in kilobase (kb). **B** Principal components plot illustrating the population structure based on the first two principal components

The genome-wide LD decay was plotted as LD (r^2^) between adjacent pairs of markers versus distance in kb which showed that average LD decay was 27.31 kb at r^2^ = 0.1 at and 9.48 kb at r^2^ = 0.2 for the panel (Fig. 1A). PCA using 214,521 SNPs (MAF ≥ 0.05) revealed moderate population structure in the association panel. The first three PCs explained about 10% of the total variance. PC1 and PC2 explained 5.2% and 3.1% of variation (Fig. 1B) and partially separated the tropical/sub-tropical lines.

**Fig. 2:**
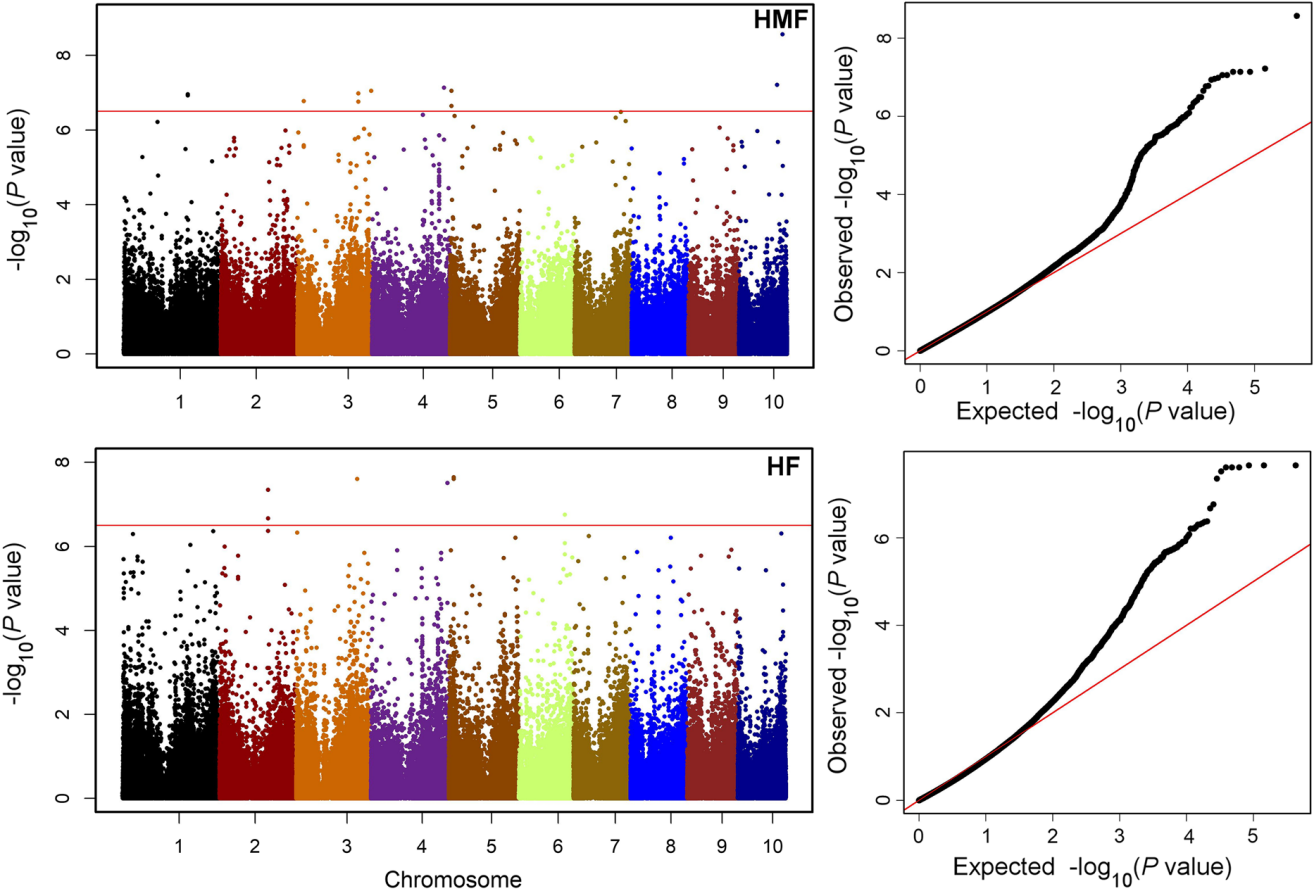
Manhattan plots based on the association scans for HMF and HF. The red horizontal line indicates the significance threshold. Quantile–quantile plots based on observed versus expected − log_10_(*P* values) are shown. (Color figure online)

**Fig. 3:**
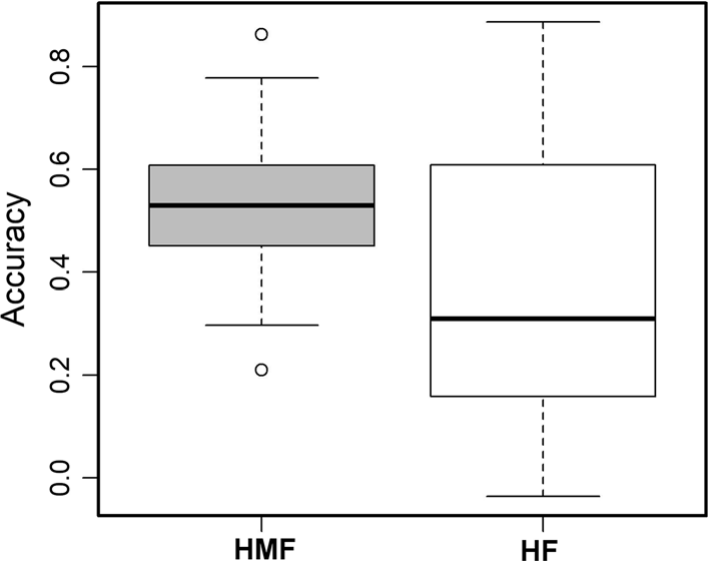
Genome-wide prediction for HMF and HF within association mapping panel based on five-fold cross-validation

Genome-wide association mapping results for both HMF and HF are presented in Tables 4 and 5 and in Manhattan plots (Fig. 2). Quantile–quantile plots of *P* values comparing the uniform distribution of the expected − log10 *p* value to the observed − log10 *p* value for HMF and HF traits are shown in Fig. 2. For HMF, we detected eight significant markers–trait associations (Table 4). These significantly associated SNPs individually explained 12 to 15% of the total phenotypic variance and together explained 34% of the total phenotypic variance for HMF. Among these eight significantly associated SNPs, S10_136007575 and S10_118961684 on chromosome 10 appear to be the most important SNPs for HMF. For HF, a set of 11 significant SNPs distributed across six different chromosomes were identified which individually explained 10 to 14% of the total phenotypic variance (Table 5). S5_15547005 and S5_15547052 on chromosome 5 and S3_189463573 on chromosome 3 were found to be the most significantly associated SNPs for HF. Comparison of the significant SNPs for the two traits revealed one consistent genomic region on chromosome 3, at around 189 Mb. We used the B73 maize genome reference sequence to identify putative candidate genes based on the SNPs significant association with HMF and HF (Tables 4, 5). From the AM panel, a set of putative candidate genes were identified; based on their functions, these can be grouped as cell development and transcription regulation related genes. Comparison of a set of 20 lines with the highest HMF (> 45%) and HF (> 15%) against the same number of lines, which had low HMF and HF in the panel, revealed a pattern of increase in the favorable allele for the selected HMF and HF-associated SNPs. Genome-wide prediction with five-fold cross validation was applied on the BLUEs of HMF and HF within association mapping panel. For HMF, the prediction accuracy was 0.53, whereas for HF prediction accuracy was 0.37 (Fig. 3).

**Table 4:** Physical positions of SNPs significantly associated with HMF, and the predicted function or homology of candidate genes

SNP-name^a^	Chr	*P* values	R^2^	MAF	Minor Allele	Minor Allele effect	Putative candidate gene	Predicted function of candidate gene
S1_199485611	1	1.1028E − 07	0.12	0.28	C	− 60.3	GRMZM2G478417	D pollen mother cell meiosis stage; bZIP transcription factor
S3_20377821	3	1.6626E−07	0.12	0.08	G	− 12.2	GRMZM2G013884	Protein kinase superfamily protein
S3_189360474	3	1.0404E−07	0.14	0.41	G	− 60.4	GRMZM2G113397	Unknown
S3_229865961	3	8.8572E−08	0.13	0.35	T	4.23	GRMZM2G075884	Protein serine/threonine kinase activity
S4_223079313	4	7.3057E−08	0.12	0.06	G	− 73.6	GRMZM2G041530	GDSL-like lipase/acylhydrolase activity
S5_5056806	5	8.9144E−08	0.13	0.28	T	− 55.5	GRMZM2G336783	ZIP metal ion transporter
S10_118961684	10	6.0683E−08	0.12	0.23	G	− 58.1	GRMZM2G125436	Serine-type peptidase activity
S10_136007575	10	2.7204E−09	0.15	0.19	A	− 63	GRMZM2G397684	Zinc ion binding, protein binding
Total R^2^			0.34					

*MAF* minor allele frequency; R^2^ represents proportion of phenotypic variance explained by SNP

^a^The exact physical position of the SNP can be inferred from marker’s name, for example, S2_211771737: chromosome 2; 211,771,737 bp

**Table 5:** Physical positions of SNPs significantly associated with HF, and the predicted function or homology of candidate genes

SNP-name^a^	Chr	*P* values	R^2^	MAF	Minor Allele	Minor Allele effect	Putative candidate gene	Predicted function of candidate gene
S1_281591014	1	4.3509E−07	0.11	0.06	A	− 43.7	GRMZM2G145017	Unknown
S2_150803630	2	4.2395E−07	0.11	0.13	C	2.31	GRMZM2G094535	Unknown
S2_150803679	2	4.4861E−08	0.12	0.13	G	2.78		
S2_150803892	2	2.1394E−07	0.10	0.12	C	0.15		
S3_189463573	3	2.4692E−08	0.14	0.21	C	− 41.5	GRMZM5G867518	Ribosomal protein S25 family protein
S4_237952441	4	3.0496E−08	0.13	0.07	A	− 31.7	GRMZM2G139372	bHLH-transcription factor
S5_15463392	5	2.4791E−08	0.13	0.08	C	− 44.3	GRMZM2G038801	Heat shock protein binding
S5_15547005	5	2.2314E−08	0.12	0.06	A	− 44.6	GRMZM2G112149	5-methyltetrahydropteroyltriglutamate-homocysteine S-methyltransferase activity
S5_15547052	5	2.2314E−08	0.12	0.06	T	− 44.6		
S6_142099026	6	1.7386E−07	0.11	0.08	G	13.88	AC215201.3_FG008	Transcription regulation
S6_164263295	6	1.6801E−07	0.14	0.08	A	− 62.1	GRMZM2G023133	Cytochrome b561/ferric reductase transmembrane protein
Total R^2^			0.35					

*MAF* minor allele frequency, *R*^2^ represents proportion of phenotypic variance explained by SNP

^a^The exact physical position of the SNP can be inferred from marker’s name, for example, S2_211771737: chromosome 2; 211,771,737 bp

## Discussion

### Variation for HMF and HF in tropical/subtropical inbred lines

Efficiency of maize in DH line production process can be greatly enhanced and the cost of DH line production may be reduced substantially if spontaneous HMF and HF could be increased and introduced into a wide array of breeding germplasm. However, there were very few systematic studies that identified germplasm with high spontaneous HMF and HF for potential use in tropical maize breeding programs. Studying HMF and HF in a large number of germplasm entries is cumbersome as several resource-intensive steps are involved, including haploid induction in a broad-based germplasm, accurate identification of sufficient number of haploids, and large field trials to assess HMF and HF. The present study is the first systematic study of HMF and HF in tropical maize germplasm, including identification of promising sources for HF, and genomic regions influencing the trait.

This study revealed that maternal haploids from ~ 75% of the tropical/subtropical inbred lines generally demonstrate poor spontaneous chromosome doubling, with less than 5% HF. Using artificial chromosomal doubling methods, it is common to achieve overall HF rates > 10% (Melchinger et al. 2016). Reliance on spontaneous genome doubling for DH line production in germplasm with low HMF and HF warrants production and identification of a significantly larger number of haploids. In addition, large numbers of haploids must to be grown and monitored in comparison to artificial chromosome doubling protocols. This significantly increases the costs of DH line production from such germplasm, especially in locations where the cost of labor is high. Hence, it may not be currently pragmatic to depend on spontaneous chromosome doubling for DH line production in a substantial proportion of tropical/subtropical maize germplasm. Interestingly, in this study we found 11 promising inbred lines with > 50% HMF and > 10% HF which could be potentially used as sources for improving these traits. Three lines (DTMA-159, DTMA-59, and CML364) were found particularly promising, showing > 50% HMF and ~ 50% HF. The results corroborate previous observations that germplasm with high rates of spontaneous chromosome doubling exist in elite maize germplasm, and that it may not be necessary to look for high haploid fertility in poorly adapted genetic resources (Geiger and Schönleben 2011; Kleiber et al. 2012).

In addition, significant genetic variances and high heritabilities were observed for both HMF and HF in tropical/subtropical germplasm used in this study, similar to what was observed in temperate maize germplasm (Geiger and Schönleben 2011; Kleiber et al. 2012; Wu et al. 2017). Genotype x environment interaction variance was significant for HMF indicating the role of non-additive genetic effects in the expression of HMF. Similar significant environment effects were also reported for HMF (Ma et al. 2018). Higher heritabilities indicate the amenability of these traits for improvement in breeding germplasm (Geiger and Schönleben 2011; Kleiber et al. 2012). Previous studies showed that HMF could be increased significantly with recurrent selection in temperate maize germplasm (Cai et al. 2017; Zabirova et al. 1993). Hence, the inbred lines with high HMF and HF identified in this study could be further improved and can be used as trait donors for introgression into other elite germplasm, thereby reducing reliance on artificial chromosome doubling and increasing the efficiency of DH line development in maize breeding programs.

This study also revealed significant correlations between haploid fertility traits with other agronomic traits. Significantly positive correlations were observed for fertility traits with plant height, ear height and number of tassel branches. A previous study using haploids from tropical maize landraces, OPVs and single crosses also reported significant correlations between plant height, ear height and HF (Kleiber et al. 2012). It was also observed that fertility in haploids derived from elite lines was higher compared to non-elite lines, and elite maize crosses compared to landraces (Kleiber et al. 2012). Together, these observations indicate that genotypes with vigorous haploids may have higher potential for spontaneous chromosome duplication. HMF and HF are tightly correlated as HMF is critical for HF. HIR is not correlated with haploid fertility and is most likely governed by independent set of genes, as was also indicated in previous studies (Barret et al. 2008; Ma et al. 2018; Prigge et al. 2012; Ren et al. 2017).

### Genetic architecture of HMF and HF

The genetic architecture of HMF has been reported in two biparental population-based QTL studies and one on testcross population-based GWAS (Ma et al. 2018; Ren et al. 2017; Yang et al. 2019). Studies using biparental populations focused particularly on temperate germplasm whereas the recent association mapping study based on large number of diverse lines, including both tropical and temperate germplasm, revealed some consistent genomic regions for HMF (Ma et al. 2018). In this study we evaluated HMF and HF on haploids produced from tropical/subtropical inbred lines.

For HMF, the most significant association found on chromosome 10 (SNP S10_118961684) was consistent with earlier GWAS study on testcross populations (Ma et al. 2018). Yang et al. (2019) reported *qHMF*3c in a biparental population and Ren et al. (2017) observed *qhmf*2 on bin 3.06 which are in consistent with the two SNPs S3_189360457 and S3_189360457 identified in the current study. These consistently identified genomic regions on chromosomes 3 and 10 are potential sources for favorable alleles of HMF. Additional significant markers identified in this study on chromosome 1, 3, 4, 5, and 10 seem to be novel and were not found in the previous QTL or association mapping studies. Overall, HMF is a complex trait controlled by a few major effect QTL (Ren et al. 2017; Yang et al. 2019) and many minor effect QTL. A few QTL detected in this study are consistent across genetic backgrounds and a few are specific to the current study can be used as potential sources for improving HMF in diverse breeding materials by rapid-cycle recurrent selection particularly for consistent major effect QTL.

The full potential of spontaneous chromosome doubling will be realized only when the germplasm with high HMF also results in high HF. Hence it is also important to understand the genetic architecture of HF. This is the first study in temperate or tropical maize germplasm reporting the genetic architecture of HF. Marker S5_15547005 on chromosome 5 was most significantly associated SNP with HF, explaining 12% of phenotypic variation. Comparison of QTL for HMF and HF revealed colocations of QTL from previous studies (Ma et al. 2018; Ren et al. 2017; Yang et al. 2019). Significant SNP on chromosome 1 (S1_281591014) was co-located with previously reported HMF QTL *qhmf1* which positioned between 286 and 296 Mb (Ren et al. 2017). The position of the significant marker S1_281591014 was also within the interval of the QTL flanked by umc1222 and bnlg1007 SSRs reported for HMF by Yang et al. (2019).

The QTL *qhmf2* reported by Ren et al. (2017) ranged between 136 and 198 Mb on chromosome 3. We found one significant marker S3_189463573 for HF in the same region and this region was also reported by association studies as an important genomic region for HMF (Ma et al. 2018). Another QTL *qhmf*4 is reported on chromosome 6 which is also fine mapped to 800 kb region (Ren et al. 2017). Even though we were not able to find any markers in this region for HMF, we found two SNPs for HF (S6_142099026 and S6_164263295) overlapping with the genomic region which supports the relevance of these markers for HMF and for HF. This high correlation between HMF and HF (r = 0.77, *P *< 0.01) and high co-localization of QTL for HF and HMF warrants further research to understand whether both traits are controlled by same genes or different genes and their interactions which pave the way for developing lines improved for both HMF and HF. Overall, HF is a complex polygenic trait governed by many moderate effect QTL.

GWAS revealed a set of putative candidate genes identified on chromosome 1, 3, 4, 5 and 10 for HMF; these were primarily involved in cell development or cell-to-cell transport (Table 4). One of the genes (GRMZM2G478417) is annotated as being involved in pollen mother cell meiosis and may play a role on the restoration of HMF. Another SNP (S4_223079313) present within the gene (GRMZM2G041530) is involved in GDSL-like lipase/acylhydrolase activity which has a role in seed development. For HF, three significant SNPs were found in the gene GRMZM2G094535 located at 150 Mbp in chromosome 2, but at present the gene function is unknown. Further, two SNPs at 15 Mbp in chromosome 5 are within the gene GRMZM2G112149 which is associated with 5-methyltetrahydropteroyltriglutamate-homocysteine S-methyltransferase activity. We failed to link the function of this gene with HF but it has a putative role in drought tolerance (Wang et al. 2016).

Genomic predictions revealed moderate accuracy for HMF and HF which supported their quantitative nature. Compared to HMF, HF seems to be more complex as it showed lower prediction accuracy. This lower prediction accuracy is also possibly due to missing of all favorable alleles for HF in most of the lines in the present panel, as evident by > 40% of lines in the panel showing < 1% of HF. Interestingly, for both the traits, we found only 2 to 3% of the lines carrying most of the favorable alleles. The most significant markers which are also consistent with previous studies could act as potential sources for introgression into other elite lines lacking the trait.

### Probable mechanism of spontaneous genome doubling and restoration of fertility in maize haploids

Even though mechanisms of spontaneous chromosomal doubling are not yet fully understood, similar mechanisms may be responsible for both artificial and spontaneous chromosomal doubling. Meiotic restitution is one of the mechanisms that can result in incomplete meiotic cell division, thereby leading to unreduced male gametes (Adams and Wendel 2005; Liu et al. 2018; Mason and Pires 2015). In maize haploids, Shamina and Shatskaya (2011) reported two different mechanisms for meiotic restitution in pollen mother cells. One mechanism involves spindle deformation leading to asymmetric incomplete cytokinesis, and the second mechanism results from inability to form the daughter cell membranes resulting in a cell with two nuclei. Gayen and Sarkar (1995, 1996) observed extensive cytomixis in colchicine-treated maize haploids, where intercellular migration of nuclei happened, and cells formed into two to six cell clusters. In such haploids with extensive cytomixis, 15- to 20-fold higher pollen fertility was observed. It is possible that cytomixis can happen during restoration of natural fertility in haploids. The mechanism(s) behind spontaneous chromosomal doubling needs to be further explored.

## Conclusion

Artificial chromosomal doubling protocols requires significant financial resources for establishing and operating facilities for germination, recovery of seedlings, chemical treatments and waste disposal. In addition, all these steps demand significant human resources. These challenges limit the adoption of DH technology by small maize breeding programs especially in the developing world. Enhancing the natural fertility of haploids in breeding germplasm will make direct planting of haploids in the fields using planting equipment possible, resulting in great reductions in resources required for the chromosomal doubling process. In addition, higher levels of fertility in haploids allows phenotypic and molecular marker-based selection approaches to be implemented at the haploid stage.

## Electronic supplementary material

Below is the link to the electronic supplementary material.

Haploid/diploid identification based on plant characteristics. Haploid and F1 plants (diploids) derived from the induction cross of the same inbred were shown at 28 days after planting (JPEG 336 kb)

Distribution of phenotypic performance of tropical inbred lines in association panel for haploid male fertility and haploid fertility across environments (JPEG 316 kb)

Summary of the heterogeneity, minor allele frequency (MAF) and proportion of missing SNPs of 214,520 selected SNPs. Chromosome assignments are indicated. The heterogeneity, MAF, and percentage of missing value are shown in left on y-axis, and the number of markers for each chromosome was shown in right on y-axis (JPEG 70 kb)
