# Supplementary figures and images for: Genome-wide association study to identify genomic regions influencing spontaneous fertility in maize haploids
Source: Euphytica. 2019 Jul 8;215(8):138. doi: 10.1007/s10681-019-2459-5 (PMC6647887; doi:10.1007/s10681-019-2459-5)

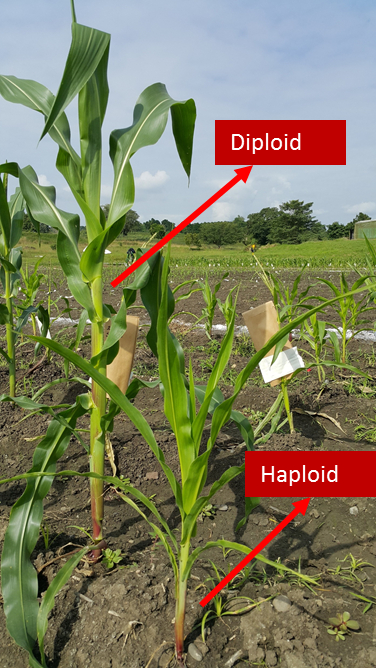

Supplement: Supplementary file 1 — Haploid/diploid identification based on plant characteristics. Haploid and F1 plants (diploids) derived from the induction cross of the same inbred were shown at 28 days after planting (JPEG 336 kb) [file 10681_2019_2459_MOESM1_ESM.jpg]

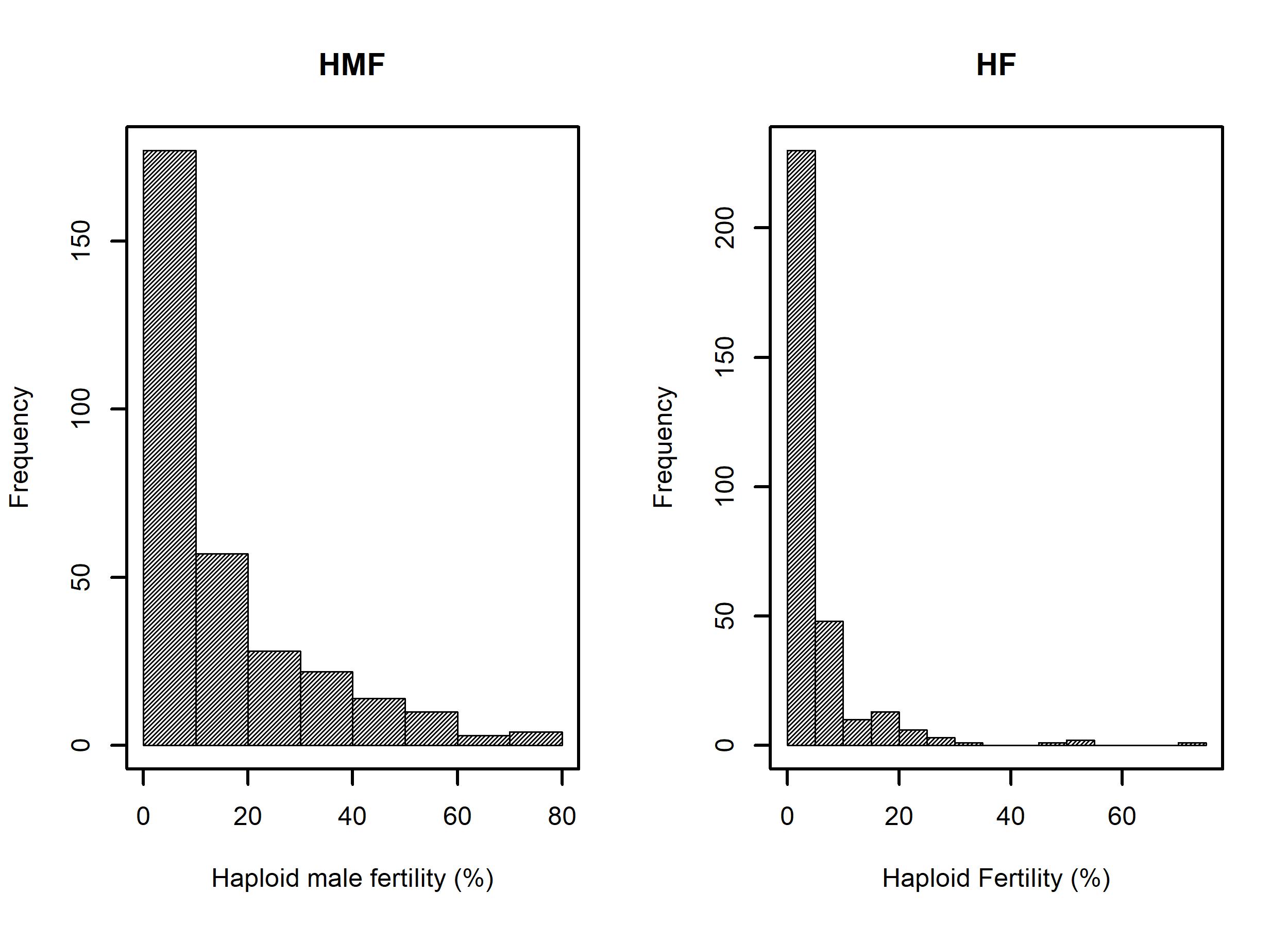

Supplement: Supplementary file 2 — Distribution of phenotypic performance of tropical inbred lines in association panel for haploid male fertility and haploid fertility across environments (JPEG 316 kb) [file 10681_2019_2459_MOESM2_ESM.jpeg]

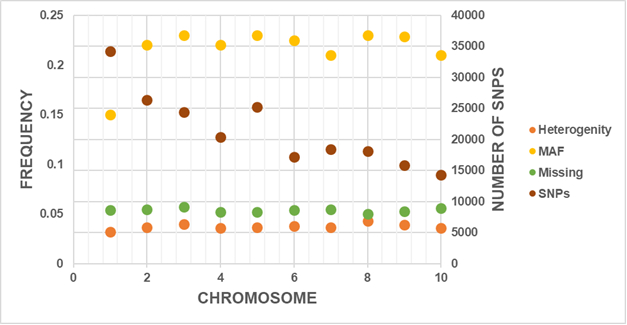

Supplement: Supplementary file 3 — Summary of the heterogeneity, minor allele frequency (MAF) and proportion of missing SNPs of 214,520 selected SNPs. Chromosome assignments are indicated. The heterogeneity, MAF, and percentage of missing value are shown in left on y-axis, and the number of markers for each chromosome was shown in right on y-axis (JPEG 70 kb) [file 10681_2019_2459_MOESM3_ESM.jpg]
